# Bronchial branching patterns and volumetry in the right upper lobe: impact on segmentectomy planning

**DOI:** 10.1093/icvts/ivad136

**Published:** 2023-08-17

**Authors:** Kentaro Miura, Takashi Eguchi, Shogo Ide, Shuji Mishima, Shunichiro Matsuoka, Tetsu Takeda, Kazutoshi Hamanaka, Kimihiro Shimizu

**Affiliations:** Division of General Thoracic Surgery, Department of Surgery, Shinshu University School of Medicine, Matsumoto, Japan; Division of General Thoracic Surgery, Department of Surgery, Shinshu University School of Medicine, Matsumoto, Japan; Division of General Thoracic Surgery, Department of Surgery, Shinshu University School of Medicine, Matsumoto, Japan; Division of General Thoracic Surgery, Department of Surgery, Shinshu University School of Medicine, Matsumoto, Japan; Division of General Thoracic Surgery, Department of Surgery, Shinshu University School of Medicine, Matsumoto, Japan; Division of General Thoracic Surgery, Department of Surgery, Shinshu University School of Medicine, Matsumoto, Japan; Division of General Thoracic Surgery, Department of Surgery, Shinshu University School of Medicine, Matsumoto, Japan; Division of General Thoracic Surgery, Department of Surgery, Shinshu University School of Medicine, Matsumoto, Japan

**Keywords:** Bronchial branching pattern, Right upper lobe, Volumetry, Pulmonary segmentectomy

## Abstract

**OBJECTIVES:**

The use of segmentectomy is expected to increase. However, understanding of the segmental bronchial branching is limited. Herein, we aimed to investigate bronchial branching pattern complexity and segmental volumetry of the right upper lung lobe to develop an accurate understanding of segmental anatomy and contribute to the advancement of safe and efficient lung segmentectomy.

**METHODS:**

We evaluated chest computed tomography scans of 303 patients and categorized the branching of segmental bronchi (segment 1, apical; segment 2, posterior; and segment 3, anterior) into 4 major types (typical trifurcated, bifurcated non-defective, bifurcated defective and atypical trifurcated) and 11 subtypes. Segmental volumetry was performed to determine the predominant segment in each case (volume difference <5% was considered equal). Branching complexity was evaluated separately for volumetry-predominant and volumetry-non-predominant segments.

**RESULTS:**

Trifurcated non-defective was the most frequent branching type (64.4%), followed by bifurcated non-defective (22.1%), bifurcated defective (8.6%) and trifurcated half-defective (4.0%). In terms of segmental volumetry, most cases had a one-segment-predominant distribution (71%) and only 5% of cases had equal distribution (segment 1 = segment 2 = segment 3). More than half of the cases had a segment 3-predominant distribution (52%). Branching complexity analysis revealed that the volumetry-non-predominant segment was associated with a higher risk of complex branching patterns compared with the volumetry-predominant segment (37% vs 19%, respectively; *P* < 0.005).

**CONCLUSIONS:**

Volumetric assessment of the right upper lobe showed a heterogeneous segmental volume distribution. Care should be taken during lung segmentectomy of the volumetry-non-predominant segments because of the high risk associated with complex bronchial branching patterns.

**Clinical trial registration:**

No. 4840.

**Figure:**
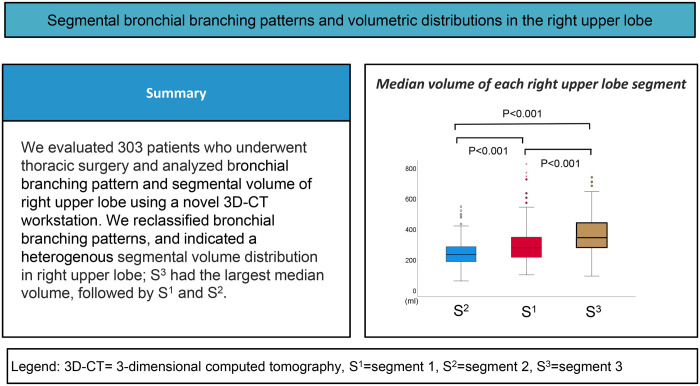

## INTRODUCTION

Segmentectomy is associated with significant preservation of pulmonary function. Therefore, it has been increasingly performed for small lung tumours, including primary lung cancer or metastatic lung tumours [1–5]. Recently, a large, multi-institutional, prospective randomized trial comparing the outcomes of lobectomy and segmentectomy for small primary lung cancers (≤2 cm) (JCOG0802/WJOG4607L) revealed that the overall survival of patients who underwent segmentectomy was significantly better than that of patients who underwent lobectomy. Despite a higher local recurrence in the segmentectomy group compared with the lobectomy group, there were no significant between-group differences in the risk of lung cancer death. More importantly, death from other cancers, respiratory disease and cerebrovascular disease occurred more frequently in the lobectomy group than in the segmentectomy group [6]. Therefore, the use of segmentectomy is expected to increase.

Preoperative prediction of resected and preserved lung volumes after lung resection is one of the most crucial functional assessments for safe surgery, and many thoracic surgeons have relied on the number of segments to be removed to estimate postoperative pulmonary function [7]. This strategy is based on the assumption that all segments have equal volumes. However, the role of segmental volumetry in these calculations has not been elucidated.

In general, lung segmentectomy is technically more challenging than lobectomy because of the complexity of the segmental bronchovascular anatomy. Although there are unlimited variations in the hilar segmental anatomy, several anatomical classifications of the lung hilum have been reported based on research using human cadaver organs [8, 9]. Our colleague demonstrated that three-dimensional computed tomography (3D-CT) imaging is useful for easy and precise measurement of anatomical branching patterns of the bronchovascular tree compared with traditional methods for studying anatomy using cadavers [10]. In addition, we developed a simplified 3D anatomical model of the right upper lobe (RUL) for appropriate intraoperative access to the intersegmental vein, which is an essential topographic landmark for lung segmentectomy [10–12]. During lung segmentectomy, surgeons should equally address the anatomical complexity and anomalies of the pulmonary artery and bronchus.

The RUL is the most lung cancer-prone lobe [13, 14], and the RUL bronchial tree is characterized by several complicated anatomical patterns (e.g. a defective branching pattern) [8, 10, 11]. In patients with a complex bronchial branching pattern, the potential risk of inappropriate segmentectomy, including excessive or insufficient resections, is high. However, understanding of the anatomical complexity of segmental bronchial branching is limited, and no studies have investigated bronchial branching patterns from the perspective of lung segmentectomy. Moreover, in previous studies, many patients showed unclassified branching patterns [8, 10, 11], which also negatively affects the approach to adequate segmentectomy.

To address the above-mentioned issues and knowledge gaps, in this study, we investigated the segmental bronchial tree of the RUL using 3D-CT. We specifically focused on segmental volumetry to determine potential variability in segmental volumes and evaluated the anatomical complexity of bronchial branching patterns to (i) develop a more thorough, comprehensive, and clinically useful anatomical classification; (ii) analyse segmental volumetry; and (iii) evaluate bronchial branching complexity from the perspective of lung segmentectomy. We believe that this investigation will contribute to the advancement of safe and effective approaches to lung segmentectomy.

## MATERIALS AND METHODS

### Ethical statement

This study was approved by the Institutional Review Board of Shinshu University Hospital (No. 4938). Due to the retrospective nature of the study, written consent was not necessary.

### Study cohort

In this retrospective study, we investigated consecutive patients who underwent thoracic surgery at Shinshu University Hospital between January 2019 and August 2021 (*n* = 574). Patients with a history of pulmonary resection (*n* = 20), those with pulmonary inflammatory changes in the RUL (*n* = 8) and those without available preoperative thin-slice CT images (0.63-mm-thick images) (*n* = 243) were excluded. A total of 303 patients were included in this study.

### Three-dimensional computed tomography reconstruction

We utilized a novel 3D-CT workstation (REVORAS, Ziosoft, Tokyo, Japan) for 3D-CT reconstruction and subsequent volumetry (details are described in the Supplementary Material).

### Segmental branching pattern of the right upper lobe bronchus

Branching patterns were categorized into 4 major types: (i) typical trifurcated, (ii) bifurcated non-defective, (iii) bifurcated defective and (iv) atypical trifurcated. The non-defective type was defined as branching patterns with all subsegmental bronchi arising from their corresponding segmental bronchial branches (e.g. B^1^a and B^1^b arising from B^1^). The defective type was defined as branching patterns with 2 subsegmental bronchial branches arising from 2 separate non-corresponding segmental bronchial branches (e.g. B^1^a from B^2^ and B^1^b from B^3^).

We created several rules with technical terminology to develop the branching patterns (details are described in the Supplementary Material). There were 11 branching patterns classified into 4 major types: (i) ‘typical trifurcated type’ including <B^1^/B^2^/B^3^>; (ii) ‘bifurcated non-defective type’ including <B^1^ + B^2^/B^3^>, <B^1^ + B^3^/B^2^> and <B^1^/B^2^ + B^3^>; (iii) ‘bifurcated defective type’ including <BX^1^a + B^2^/BX^1^b + B^3^>, <B^1^ + BX^2^a/B^3^ + BX^2^b> and <B^1^ + BX^3^b/B^2^ + BX^3^a>; and (iv) ‘atypical trifurcated type’ including <B^1^a/B^2^/BX^1^b + B^3^>, <BX^1^a + B^2^/B^1^b/B^3^>, <B^1^ + BX^2^a/B^2^b/B^3^> and <B^1^/B^2^a/BX^2^b + B^3^>. In addition to the 4 major branching types, we defined a variant branching pattern as the *displaced type* in which a segmental or subsegmental branch arises from the trachea or bronchus other than the RUL bronchus (Supplementary Material, Fig. S1).

We summarized the data on branching patterns in our cohort and compared them with those from previous studies (Supplementary Material, Table S1) [10].

### Segmental volumetric analysis of the right upper lobe

**Figure 1:**
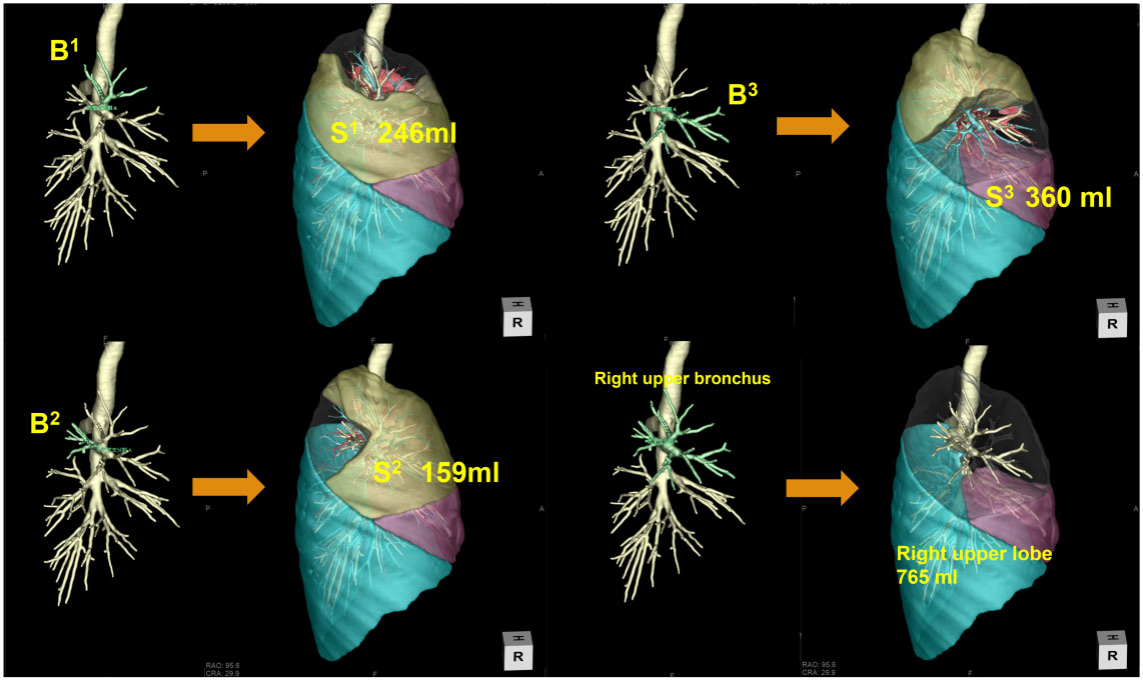
Actual method for segmental volumetry of the right upper lobe. First, bronchial bifurcation in the right upper lobe is identified. Subsequently, each target bronchus is selected, and the segmental volume is automatically calculated.

The volume of each segment was measured semi-automatically by selecting the index segmental bronchial branch on the 3D workstation (Fig. 1). If there was more than 1 subsegmental branch arising from a different bronchus, we selected all branches that entered the index segment to measure segmental volume.

We measured the distribution of segmental volumes to determine the volumetric relationships between each segment, including equal (=), larger (>) and smaller (<) relationships. An equal relationship between segments (e.g. S^1^ = S^2^) was defined as a difference in volumetric distribution of ≤5%.

We classified 3 major volumetric relationships as follows: ‘equality relationship’ (S^1^ = S^2^ = S^3^), ‘one-segment-predominant relationship’ [S^1^ predominant, S^2^ predominant and S^3^ predominant; each segment-predominant relationship includes 3 patterns (e.g. S^1^ predominant: S^1^ > S^2^ > S^3^, S^1^ > S^3^ >S^2^, or S^1^ > S^2^ = S^3^)] and ‘other relationship’ [including a pattern with 2 equally larger segments and 1 smaller segment (e.g. S^1^ = S^2^ > S^3^)].

### Segmentectomy-specific complexity based on bronchial branching patterns

Assuming there are segments α and β, and there is a patient with a small lung tumour in segment α (S^α^) who is scheduled for S^α^ segmentectomy, a corresponding segmental bronchus (B^α^) should be divided during surgery. When there are 2 subsegmental branches (B^α^a and B^α^b) arising from 2 separate bronchi [defective branching pattern (B^α^/B^β^ + BX^α^b)] or when B^α^ is a single branch but arises from a common branch with another segmental bronchus (B^β^), which should be preserved [common branching due to a bifurcated branching pattern (B^α^ + B^β^)], the potential risk of inappropriate surgery, including insufficient or excessive resection, is high. Therefore, the complexity of the B^α^ branching pattern will affect operative difficulty during S^α^ segmentectomy.

In this study, we investigated the incidence of complex branching patterns in each segmental bronchus during an assumed, corresponding segmentectomy. Assuming S^α^ segmentectomy is planned (S^β^ and S^γ^ are segments to be preserved), a complex branching pattern of B^α^ was defined as follows: (i) ‘defective branching pattern’, 2 separate subsegmental branches (B^α^a and B^α^b) arising from 2 separate bronchi (defective pattern [B^β^ + BX^α^a/B^γ^ + BX^α^b] or [B^α^a/B^β^ + BX^α^b/B^γ^]); (ii) ‘common branching pattern’, a segmental bronchial branch (B^α^) arising from a common bronchus (B^α^ + B^β^), in which B^β^ should be preserved. In contrast, a simple branching pattern of B^α^ was defined as cases with B^α^ that arises directly from the lobar bronchus.

We hypothesized that an uneven volumetric distribution could be related to the bronchial branching pattern, based on our surgical experience with lung segmentectomy. Therefore, we focused on the potential relationship between volumetric predominance and branching complexity and compared the incidence of complex branching patterns between segmentectomies of volumetry-predominant and volumetry-non-predominant segments.

### Statistical analysis

All statistical analyses were performed using SPSS version 27 (Chicago, IL, USA). Details are described in the Supplementary Material.

## RESULTS

### Patient characteristics and bronchial branching patterns

**Figure 2:**
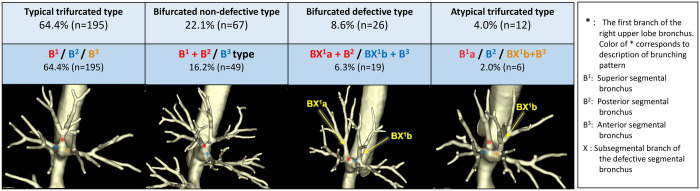
Major classification and representative 3D image of right upper lobe bronchus. *The first branch of the right upper lobe bronchus.

Table 1 shows the characteristics and bronchial branching patterns of the study participants. We investigated 574 consecutive patients who underwent thoracic surgery. Patients with a history of pulmonary resection (*n* = 20), those with pulmonary inflammatory changes in the RUL (*n* = 8) and those without available preoperative thin-slice CT images (*n* = 243) were excluded. Eventually, a total of 303 patients were included in this study. Figure 2 shows a representative 3D image of the 4 major classifications of RUL bronchus. The detailed classifications are shown in Supplementary Material, Figs S1 and S2. The proportions for each branching pattern were as follows:

**Table 1:** Patient characteristics and branching patterns of the right upper lobar bronchus

	*N* = 303
Age (years)	72 (25–94)
Sex	
Male	138 (46%)
Female	165 (54%)
Smoking status	
Never	161 (53%)
Former/current	142 (47%)
Pulmonary function test	
FVC (l)	3.1 (1.2–5.4)
%FVC (%)	107 (53–171)
FEV1.0 (l)	2.7 (1.1–4.3)
FEV1.0/FVC (%)	77 (35–97)
Diagnosis	
Primary lung cancer	230 (76%)
Metastatic lung tumour	49 (16%)
Mediastinal tumour	12 (4%)
Others	12 (4%)
Bronchial branching type	
Typical trifurcated type	
B^1^ + B^2^ + B^3^	195 (64%)
Bifurcated non-defective type	
B^1^ + B^2^/B^3^	49 (16%)
B^1^ + B^3^/B^2^	10 (3%)
B^2^ + B^3^/B^1^	8 (3%)
Bifurcated defective type	
BX^1^a + B^2^/BX^1^b + B^3^	19 (6%)
B^1^ + BX^2^a/B^3^ + BX^2^b	5 (2%)
B^1^ + BX^3^b/B^2^ + BX^3^a	2 (0.7%)
Atypical Trifurcated type	
B^1^a/B^2^/BX^1^b + B^3^	6 (2%)
BX^1^a + B^2^/B^1^b/B^3^	1 (0.3%)
B^1^ + BX^2^a/B^2^b/B^3^	3 (1%)
B^1^/B^2^a/BX^2^b + B^3^	2 (0.7%)
Displaced type	
B^1^ displaced	1 (0.3%)
B^2^ displaced	0
B^3^ displaced	2 (0.7%)

Data are presented as median (interquartile range) or number (%).

B^1^: apical branch; B^2^: posterior branch; B^3^: anterior branch; BX: defective bronchial branch; FEV1.0: forced expiratory volume in 1 second; FVC: forced vital capacity.

- Typical trifurcated type (B^1^/B^2^/B^3^): 64.4% (*n* = 195).
- Bifurcated non-defective type: 22.1% (*n* = 67) [B^1^ + B^2^/B^3^ type: 16.2% (*n* = 49), B^1^ + B^3^/B^2^ type: 3.3% (*n* = 10), B^1^/B^2^ + B^3^ type: 2.6% (*n* = 8)].
- Bifurcated defective type: 8.6% (*n* = 26) [BX^1^a + B^2^/BX^1^b + B^3^ type: 6.3% (*n* = 19), B^1^ + BX^2^a/B^3^ + BX^2^b type: 1.7% (*n* = 5), B^1^ + BX^3^b/B^2^ + BX^3^a type: 0.7% (*n* = 2)].
- Atypical trifurcated type: 4.0% (*n* = 12) [B^1^a/B^2^/BX^1^b + B^3^ type: 2.0% (*n* = 6), BX^1^a + B^2^/B^1^b/B^3^: 0.3% (*n* = 1), B^1^ + BX^2^a/B^2^b/B^3^ type: 1.0% (*n* = 3), B^1^/B^2^a/BX^2^b + B^3^ type: 0.7% (*n* = 2)].
- Displaced type: 1.0% (*n* = 3).

### Segmental volumetric analysis of the right upper lobe—predominant and non-predominant segments

**Figure 3:**
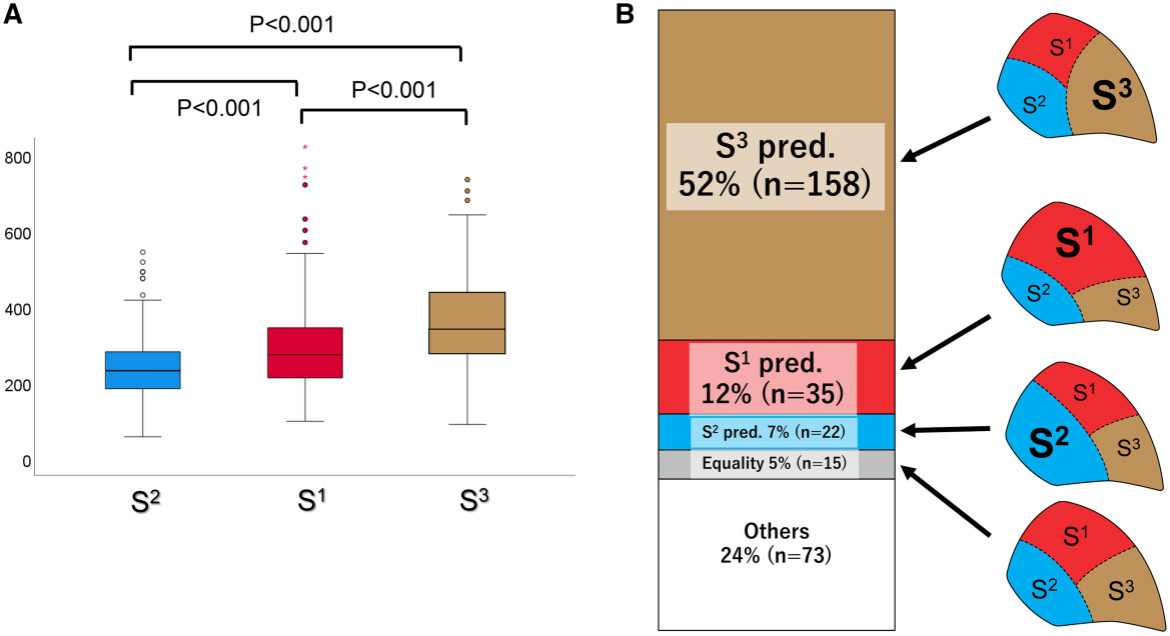
(**A**) Median volume of each right upper lobe segment. (**B**) Volume distribution of the 3 segments and their frequencies in the right upper lobe.

Figure 3A shows the mean volumes of the S^1^, S^2^ and S^3^ segments in the RUL of the 303 patients. S^3^ had the largest median volume [342.6 ml; interquartile range (IQR), 280.2–438.7 ml], followed by S^1^ (median volume, 273.6 ml; IQR, 211.5–344.9 ml) and S^2^ (median volume, 231.2 ml; IQR, 182.2–281.8 ml) (*P* < 0.001). Figure 3B shows the volume distribution pattern in the RUL, with the following frequencies:

- Equality relationship (S^1^=S^2^=S^3^): 5% (*n* = 15).
- Segment-predominant relationship: 71% (*n* = 215) [S^1^ predominant: 12% (*n* = 35), S^2^ predominant: 7% (*n* = 22), S^3^ predominant: 52% (*n* = 158)].
- Other relationship: 24% (*n* = 73).

Of the cases, 10.8% (*n* = 32) had an S^3^ volume exceeding 50% of the RUL (S^3^>S^1^ + S^2^ pattern; termed ‘mega S^3^’). Supplementary Material, Figure S3 shows the cases of mega S^3^, which occupied 61.6% of the RUL. A comparison of the predicted segmental volume in the RUL between the conventional method based on the number of segments or subsegments, and the volumetric analysis in the current study by a novel 3D-CT workstation has also been presented. In this case, when calculated using the conventional method, the volume of S1 and S2 was overestimated and the volume of S3 was underestimated when compared to those calculated based on 3D-CT volumetry. Table 2 shows the detailed distribution of all patterns.

**Table 2:** Distribution of each volumetric segment pattern in the right upper lobe

Predominant type	*n* (%)
Equally relationship (S^1^ = S^2^ = S^3^)	15 (5%)
One-segment-predominant relationship	215 (71%)
S^3^ (anterior segment) predominant	158 (52%)
S^3^ > S^1^ > S^2^	64
S^3^ > S^2^ > S^1^	30
S^3^ > S^1^ = S^2a^	64
S^1^ (apical segment) predominant	35 (12%)
S^1^ > S^2^ > S^3^	1
S^1^ > S^3^ > S^2^	17
S^1^ > S^2^ = S^3a^	17
S^2^ (posterior segment) predominant	22 (7%)
S^2^ > S^1^ > S^3^	3
S^2^ > S^3^ > S^1^	13
S^2^ > S^1^ = S^3a^	6
Other relationship	73 (24%)
S^1^ = S^2^ > S^3a^	21
S^1^ = S^3^ > S^2a^	46
S^2^ = S^3^ > S^1a^	6

a ‘=’ represents an equal volumetric relationship, which is defined as a volumetric difference of <5% between 2 segments.

### Complex bronchial branching pattern for each right upper lobe segmentectomy according to volumetric predominance

Table 3 shows the incidence of bronchial branching patterns based on volumetry-predominant segmental patterns. In the S^3^-predominant type (*n* = 158), 22 (14%) cases had a B^3^ complex bronchial pattern (BX^1^a + B^3^/BX^1^b/B^2^ type: 3, B^1^/BX^2^a/BX^2^b + B^3^ type: 1, B^1^ + B^3^/B^2^ type: 1, B^1^/B^2^ + B^3^ type: 4, BX^1^a + B^2^/BX^1^b + B^3^ type: 11, B^1^ + BX^2^a/B^3^ + BX^2^b type: 1, displaced B^3^: 1). In the S^1^-predominant type (*n* = 35), 10 (29%) cases had a B^1^ complex bronchial pattern (BX^1^a + B^3^/B^1^b/B^2^ type: 2, B^1^ + BX^2^a/B^2^b/B^3^ type: 1, B^1^/B^2^a/BX^2^b + B^3^ type: 1, B^1^ + B^3^/B^2^ type: 1, BX^1^a + B^2^/BX^1^b + B^3^ type: 2, B^1^ + BX^2^a/B^3^ + BX^2^b type: 2, B^1^ + BX^3^b/B^2^ + BX^3^a type: 1). In the S^2^-predominant type (*n* = 22), 6 (27%) cases had a B^2^ complex bronchial pattern (B^1^ + BX^2^a/B^2^b/B^3^ type: 2, B^1^/B^2^a/BX^2^b + B^3^ type: 1, BX^1^a + B^2^/BX^1^b + B^3^ type: 1, B^1^ + BX^2^a/B^3^ + BX^2^b type: 2).

**Table 3:** Incidence of bronchial branching patterns based on volumetry-predominant segmental patterns

	Equality	S^1^ pred.	S^2^ pred.	S^3^ pred.	Others
	*N* = 15	*N* = 35	*N* = 22	*N* = 158	*N* = 73
Bronchial branching pattern					
Typical trifurcated					
B^1^/B^2^/B^3^	12 (80%)	20 (57%)	11 (50%)	96 (61%)	56 (77%)
Bifurcated non-defective					
B^1^+B^2^/B^3^	1 (7%)	0	0	39 (25%)	9 (12%)
B^1^+B^3^/B^2^	2 (13%)	1 (3%)	5 (23%)	1 (0.6%)	1 (1%)
B^2^+B^3^/B^1^	0	4 (11%)	0	4 (3%)	0
Bifurcated defective					
BX^1^a + B^2^/BX^1^b+B^3^	0	2 (6%)	1 (5%)	11 (7%)	5 (7%)
B^1^ + BX^2^a/B^3^ + BX^2^b	0	2 (6%)	2 (9%)	1 (0.6%)	0
B^1^ + BX^3^b / B^2^ + BX^3^a	0	1 (3%)	0	0	1 (0%)
Atypical trifurcated					
B^1^a/B^2^/BX^1^b + B^3^	0	2 (6%)	0	3 (2%)	1 (1%)
BX^1^a + B^2^/BX^1^b/B^3^		1 (3%)	0	0	0
B^1^ + BX^2^a/BX^2^b/B^3^	0	1 (3%)	2 (9%)	0	0
B^1^/BX^2^a/BX^2^b + B^3^	0	0	1 (5%)	1 (0.6%)	0
Displaced					
B^1^ displaced	0	0	0	1 (0.6%)	0
B^2^ displaced	0	0	0	0	0
B^3^ displaced	0	1 (3%)	0	1 (0.6%)	0
Segmentectomy-specific bronchial branching complexity^a^					
B^3^ complexity during S^3^ segmentectomy		Non-pred.	Non-pred.	Pred.	
Complex^a^	NA	12 (34%)	9 (41%)	22 (14%)	NA
Simple^a^	NA	23 (66%)	13 (59%)	136 (86%)	NA
B^1^ complexity during S^1^ segmentectomy		Pred.	Non-pred.	Non-pred.	
Complex^a^	NA	10 (29%)	10 (45%)	56 (35%)	NA
Simple^a^	NA	25 (71%)	12 (55%)	102 (65%)	NA
B^2^ complexity during S^2^ segmentectomy		Non-pred.	Pred.	Non-pred.	
Complex^a^	NA	8 (23%)	6 (27%)	56 (35%)	NA
Simple^a^	NA	27 (77%)	16 (73%)	102 (65%)	NA

Data are presented as number (%).

a Bronchial branching patterns in the predominant segment are defined as follows: complex, any bronchial branching pattern that requires surgical division of >1 bronchial branch or only 1 branch but requires preservation of the proximal branch(es) after the first segmental branch that belongs to another segment during segmentectomy of the index segment; simple, any bronchial branching pattern that requires surgical division of only 1 index segmental bronchus without preservation of any branches after the first segmental branch. Underlines represent cases of complex bronchial patterns in the predominant segment.

B^1^: apical branch; B^2^: posterior branch; B^3^: anterior branch; BX: defective bronchial branch; NA: not applicable; Pred.: predominant; S^X^ pred.: one-segment-predominant relationship (S^1^ predominant, S^2^ predominant, and S^3^ predominant).

**Figure 4:**
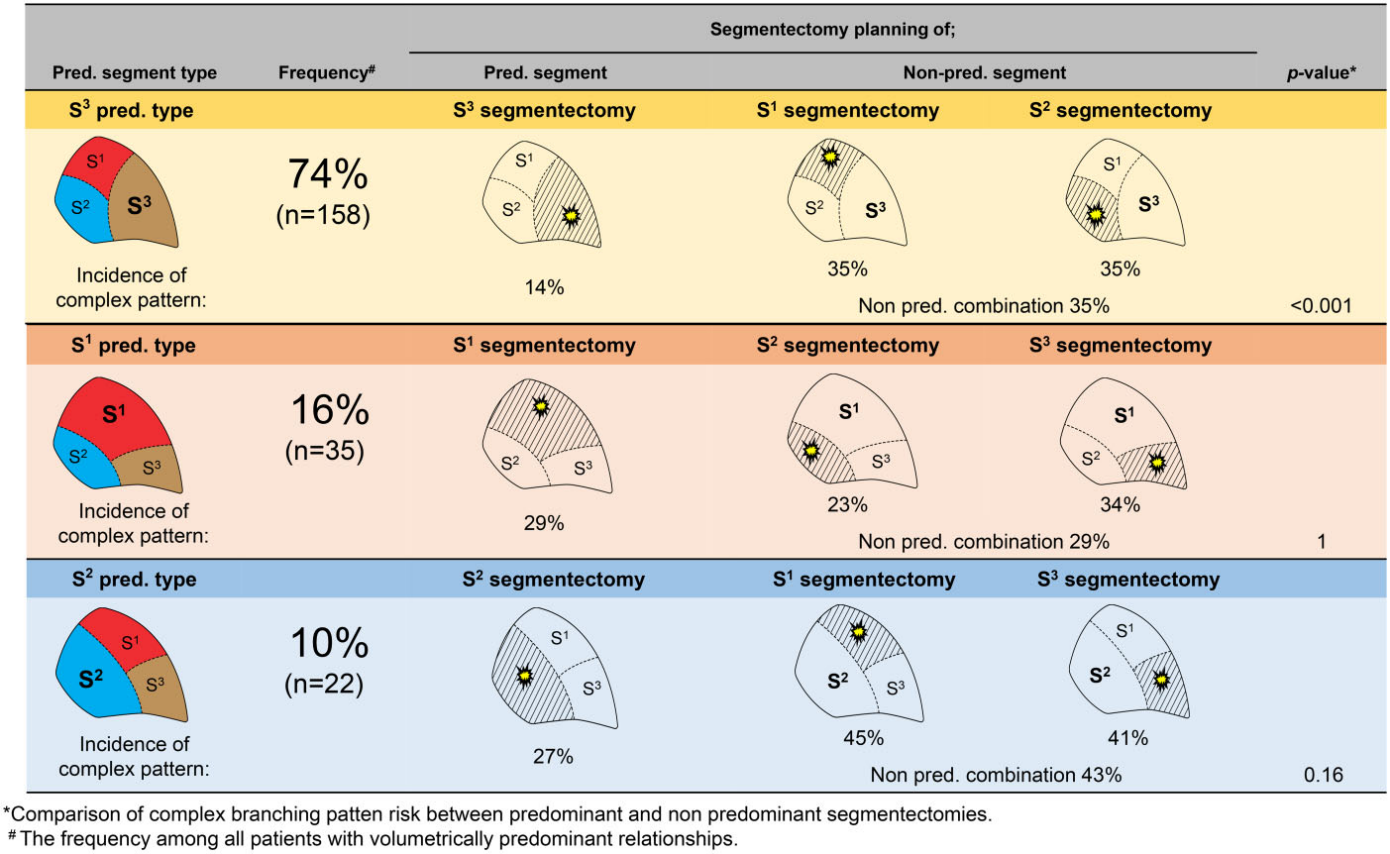
Frequencies of bronchial patterns in segmentectomy planning for each predominant pattern. In the S^3^-predominant type, the frequency of the B^3^ simple bronchial pattern is 86%, while that of the B^3^ complex pattern is 16%; in S^1^ or S^2^ segmentectomy, the frequency of B^1^ and B^2^ complex patterns is 35% (*P* < 0.001). In the S^1^-predominant type, the B^1^ complex bronchial pattern frequency is 29%; in S^2^ or S^3^ segmentectomy, the frequency of B^2^ and B^3^ complex patterns is 29% (*P* = 1). In the S^2^-predominant type, the B^2^ complex pattern frequency is 27%; in S^1^ or S^3^ segmentectomy, the frequency of B^1^ and B^2^ complex patterns is 43% (*P* = 0.16).

Figure 4 shows the frequencies of bronchial patterns in segmentectomy planning for each predominant pattern. In all patients with the one-segment-predominant relationship (*n* = 215), the incidence of complex bronchial patterns in the volumetry-predominant segment was significantly lower than that in the volumetry-non-predominant segments (18% and 35%, respectively, *P* < 0.001; Table 4). Particularly, in the S^3^-predominant type, the incidence of the B^3^ complex bronchial pattern was only 14%, whereas those of B^1^ and B^2^ were 35% and 35%, respectively (*P* < 0.001). Similarly, in the S^2^-predominant type, the incidence of the B^2^ complex bronchial pattern was higher than that of B^1^ or B^3^ (45% or 41%, respectively); however, this was not statistically significant (*P* = 0.16).

**Table 4:** Incidence of complex bronchial patterns in predominant and non-predominant segments according to volumetry-predominant segment status

Bronchial branching complexity during segmentectomy	Segmentectomy planning for		*P*-Value
	Predominant segment (*n* = 215)	Non-predominant segment^a^ (*n* = 430)	
Complex	18% (*n* = 38)	35% (*n* = 150)	<0.001
Simple	82% (*n* = 177)	65% (*n* = 280)	

a There are 2 non-predominant segments per case.

## DISCUSSION

Our topographical anatomical investigation and volumetric analysis of the RUL revealed previously unknown findings. The significance and novelty of this study are as follows: (i) our simple branching pattern classification comprising 4 major categories (i.e. typical trifurcated, bifurcated non-defective, bifurcated defective and atypical trifurcated) covers 99% of patients; (ii) volumetric analysis revealed an uneven segmental distribution, demonstrating a frequent S^3^-predominant relationship; and (iii) segmentectomy-specific bronchial branching complexity demonstrated a significant association between volumetry-non-predominant segments and a higher risk of complex branching patterns, suggesting the potential utility of volumetry in predicting surgical challenges associated with segmentectomy.

Our literature review of bronchial branching patterns revealed 3 studies describing segmental branching classifications of the RUL and their frequencies [8–10]. Among these studies, the earliest (published in 1948) investigated cadavers, whereas the most recent study investigated 3D-CT images to differentiate anatomical patterns similar to our study (published in 2015) [10]. Supplementary Material, Table S1 shows a comparison between our study and that of Nagashima *et al.* [10]. Although the distribution of the typical trifurcated category was slightly higher in our study than that in the study by Nagashima *et al.* (63% vs 44%, respectively), a significant difference between the 2 studies is that in the latter, 19% of patients were unclassified, compared with only 1% in our study. We also suggest that the significant difference in diagnostic performance between the 2 studies could be partially attributed to the difference in 3D-CT workstations. In our study, we utilized a newly developed 3D-CT workstation (REVORAS, Ziosoft, Tokyo, Japan), which enables a more vivid and clear analysis of bronchial bifurcations by measuring extrabronchial contours, compared with conventional 3D-CT workstations that measure intraluminal contours. Measurement of intraluminal contours to evaluate the bronchial tree may cause misidentification of small segmental bronchial branches, which might have affected the incidence of unclassified cases (Supplementary Material, Fig. S4).

In our study cohort, we observed 3 cases of displaced branching, which is defined as a branching pattern in which a segmental bronchus arises from a different lobar bronchus. There were 2 cases of B^3^ downwards-shifting malformation, in which B^3^ arises from the middle lobar bronchus. Yaginuma investigated the incidence of displaced bronchi using CT scans. He analysed 6072 patients and reported that displaced bronchi were more frequent in the RUL (frequency: 0.64%; *n* = 39) [15]. Another study reported an incidence of displaced bronchi in the RUL of 0.15% [16]. The incidence of the displaced pattern in our study was 1%, which is consistent with these studies.

With the global increase in high-risk elderly patients with early-stage lung cancer, the importance of predicting postoperative pulmonary function has increased. Conventional prediction of resected lung volume is based on the number of resected segments (or subsegments) [7]. Our volumetric findings suggest that the conventional prediction of postoperative pulmonary function may provide an invalid predictive value. Equally distributed RUL segments were seen in only 5% of patients, and more than two-thirds (71%) of patients had volumetric-predominant segments. In addition, 11% of patients had ‘mega S^3^’, defined as predominant S^3^ that occupies more than half of the RUL. These findings indicate that the conventional prediction method, which is based on the number of resected segments or subsegments, could lead to the underestimation or overestimation of the resected lung volume. We present a representative case of a patient with an S3 predominant distribution in Supplementary Material, Fig. 4. In this case, the predominant S3 segmentectomy removed 11% of the whole lung guided by 3D-CT volumetry. In contrast, only 4.8% was resected based on conventional methods (using subsegments). Conversely, the actual resected volume by non-predominant S1 or S2 segmentectomies was overestimated compared to the conventional method. We believe that thoracic surgeons should be aware of these findings when making decisions on surgical indications, particularly in patients who are elderly or have poor pulmonary function.

This study also provides surgeons with clinically useful information regarding the potential surgical difficulties associated with RUL segmentectomy. If a tumour develops in the non-predominant RUL segment in patients with a one-segment-predominant relationship (including 71% of patients in our cohort), the risk of having a complex bronchial branching pattern would be higher than in cases where a tumour develops in the predominant segment, particularly in the S^3^-predominant type (Fig. 4). In cases with complex bronchial branching patterns, sufficient preoperative simulation and intraoperative navigation using 3D-CT planning should be considered to avoid inappropriate segmentectomy based on misidentification of the segmental bronchial anatomy.

### Limitations

This study had several limitations. First, segmental volumetry might have been affected by patient posture (generally supine) at the time of CT scanning. Yamada *et al.* [17] analysed the differences in lung volume between the supine and standing positions using a CT scan. They showed that the volume of each lobe (except for the middle lobe) was significantly greater in the standing than in the supine position. S^2^, which is located on the dorsal side when the patient is in the supine position, could have been influenced by the effects of gravity. Second, our segmental volumetry results were based only on 3D-CT analysis. We conducted an independent study to investigate the accuracy of predicting postoperative pulmonary function based on 3D-CT volumetry, the results of which will be analysed and published later. Third, the number of patients, particularly those with minor branching patterns, was relatively small, which negatively affected the generalizability of our findings. Lastly, regarding the concept of surgical complexity, our study did not directly correlate anatomical variations with objective parameters, such as surgical time and margin distance. This limits our ability to comprehensively evaluate the relationship between anatomical variations and surgical complexity. Future studies should incorporate these objective parameters to provide more detailed insights into the impact of anatomical variations on pulmonary segmentectomy outcomes.

## CONCLUSIONS

We successfully classified all patients based on RUL segmental bronchial branching patterns. Thoracic surgeons should exercise extreme care when interpreting the relatively frequent presentation of heterogeneous volumetric distribution of RUL segments and the risk of complex bronchial branching patterns when planning RUL segmentectomy.

## Supplementary Material

ivad136_Supplementary_Data

## Data Availability

The data underlying this article cannot be shared publicly due to the privacy of individuals that participated in the study. The data will be shared on reasonable request to the corresponding author.

## ABBREVIATIONS

3D-CT: Three-dimensional computed tomography
IQR: Interquartile range
RUL: Right upper lobe
