# Supplementary material for: Bronchial branching patterns and volumetry in the right upper lobe: impact on segmentectomy planning
Source: Interdiscip Cardiovasc Thorac Surg. 2023 Aug 17;37(3):ivad136. doi: 10.1093/icvts/ivad136 (PMC10702455; doi:10.1093/icvts/ivad136)

**Supplemental materials**

**Title:** Bronchial Branching Patterns and Volumetry in the Right Upper Lobe: Impact on Segmentectomy Planning

**Authors:** Kentaro Miura*^1)^, MD, PhD, Takashi Eguchi*^1)^, MD, FACS, Shogo Ide^1)^, MD, Shuji Mishima^1)^, MD, Shunichiro Matsuoka^1)^, MD, Tetsu Takeda^1)^, MD, PhD, Kazutoshi Hamanaka^1)^, MD, PhD, and Kimihiro Shimizu^1)^, MD, PhD

*K.M. and T.E. contributed equally to this work.

**Institution:**

1. Division of General Thoracic Surgery, Department of Surgery, Shinshu University School of Medicine, Matsumoto, Japan

**Corresponding author:**

Kimihiro Shimizu

Division of General Thoracic Surgery, Department of Surgery, Shinshu University School of Medicine

3-1-1 Asahi, Matsumoto, 390-8621, Japan

Telephone: +81-263-37-3576

E-mail: [kmshimizu@gmail.com](mailto:kmshimizu@gmail.com)

**CONTENTS**

**Supplemental Text**

・3D-CT reconstruction

・Segmental branching pattern of the RUL bronchus

・Statistical analysis

・References

**Supplemental Table**

**・Supplemental Table S1.** Comparison of the distribution of the right upper lobe bronchial branching patterns between our study and a previous study.

**Supplemental Figure**

**・Supplemental Figure S1.** Detailed classifications of the RUL bronchus.

**・Supplemental Figure S2.** Branching pattern of the displaced type.

**・Supplemental Figure S3.** Illustration of a representative B^1^a/B^2^/BX^1^b+B^3^ bronchial branching pattern.

**・Supplemental Figure S4.** “Mega S3,” which occupies more than half of the right upper lobe.

**・Supplemental Figure S5.** Comparison of a 3D image and an image produced by REVORAS.

**Supplemental Video**

**・Supplemental Video S1.** A demonstration of the 3D reconstruction and volumetry.

**Supplemental Methods**

*3D-CT reconstruction*

We used two types of CT scanners during the study period: LightSpeed VCT Vision (GE Healthcare, USA; until December 2019) and Revolution CT (GE Healthcare, USA; from January 2020). All 3D-CT images were reconstructed from 0.63-mm-thick, high-resolution CT scans using volume-rendering 3D-CT reconstruction software (REVORAS, Ziosoft, Tokyo, Japan). In this software, surgeons (or radiation technologists) can easily (by an automated or semi-automated process) create the following lung surgery-specific 3D images from either contrast-enhanced or non-enhanced CT scans:

1. Pulmonary vessels (PA and PV): the software determines the center points of the PA and PV and automatically differentiates between the PA and PV to create their angiographic images separately
2. Airways (trachea and bronchus): 3D bronchography is automatically reconstructed using surface rendering technology
3. Lobes: the software develops lobar segmentation by determining interlobar fissures using the Voronoi diagram theory described below
4. Bones: 3D reconstructions of the bones in the chest, including the ribs, vertebrae, scapulae, sternum, and clavicles, were created automatically
5. Tumors: a surgeon can create a 3D reconstruction of tumors by pointing the tumors in axial, coronal, or sagittal CT images

The 3D images of the first three structures (pulmonary vessels, airways, and lobes) can be obtained by a completely automated process in which surgeons simply select a series of CT scans (“one-click 3D reconstruction”) [1]. The branching pattern of the right upper lobe segmental bronchi was then assessed. Segmental volumetric analysis of the RUL was automatically performed using the software. Figure 1 illustrates the segmental bronchial branches and corresponding volumetric measurements in a representative case. A video demonstration of the 3D reconstruction and volumetry is available in the supplemental material. For lung parenchyma segmentation, the concept of the Voronoi diagram was utilized in the software, which involves parting a plane into regions based on a set of points called seeds. For each seed, there is a corresponding region consisting of all points closer to that seed. In other words, a partition has the same distance from each seed in the neighboring regions. For lobar segmentation, a partition represents the interlobar fissure, a region as the lobe, and a seed as the peripheral bronchus [2] [3].

*Segmental branching pattern of the RUL bronchus*

We classified bronchial branching patterns based on the position of three segmental branches (B^1^ [apical branch], B^2^ [posterior branch], and B^3^ [anterior branch]) and the six subsegmental branches (B^1^a, B^1^b, B^2^a, B^2^b, B^3^a, and B^3^b). B^1–3^a represents a posterior subsegmental bronchus, and B^1–3^b represents an anterior subsegmental bronchus.

First, the axis of the airways (a line connecting the center points of the airway) was drawn automatically. Second, the distance between two branching points was measured manually at the workstation. Third, we differentiated trifurcated and bifurcated patterns based on the following rule: if the distance between two branching points was 5 mm or less, they were considered the same branch (trifurcated type); in contrast, if the distance was more than 5 mm, they were considered two different branches, in which case a bronchus between the two branching points was considered the common branch of the two segmental bronchial branches (bifurcated type).

1. A branching pattern of the segmental bronchus was described between the left and right angle brackets (e.g., <B^1^/B^2^/B^3^>).
2. The slash (/) represents the first branching point(s) after upper lobar bronchial takeoff. Therefore, only one slash is used in the brackets in cases with a bifurcated type (e.g., <B^1^/B^2^+B^3^>), whereas two slashes are used in cases with a trifurcated type (e.g., <B^1^/B^2^/B^3^>, in which three segmental branches separate at once at the first trifurcating point of the upper lobar bronchus).
3. The plus sign (+) represents the common branch of two separate segmental or subsegmental bronchi having their bifurcation at the periphery (e.g., <B^1^/B^2^+B^3^> represents the first bifurcation of B^1^ and the common branch of B^2^ and B^3^; thereafter, the further bifurcation of B^2^ and B^3^ exists at the periphery of the first bifurcation).
4. “BX” represents a defective subsegmental branch, which is defined as a subsegmental bronchus arising from a non-corresponding segmental bronchus. For example, in the case of the <B^2^+BX^1^a/B^3^+BX^1^b> pattern, an independent B^1^ branch (no corresponding segmental bronchus for B^1^a and B^1^b) is lacking; instead, a posterior subsegmental branch (BX^1^a) arises from B^2^ and an anterior subsegmental branch (B^1^b in the non-defective pattern) arises from B^3^.
5. B^1–3^a represents a posterior subsegmental branch, and B^1–3^b represents an anterior subsegmental branch.

Defining a defective bronchus is sometimes difficult. As segmental partitioning of the lung is determined by the intersegmental planes with their corresponding intersegmental veins, we utilized the location of the intersegmental veins to differentiate between a defective branch of the index segment and a branch of the adjacent segment (Supplemental Figure S2).

*Statistical analysis*

Each segmental volume was presented as the median and interquartile range (IQR) and was analyzed using Mann-Whitney U test with Bonferroni’s correction. In all analyses, statistical significance was set at p<0.05. The incidence of complex and simple bronchial patterns in predominant and non-predominant segments according to the volumetry-predominant segment status was compared using the *chi-squared* test. A difference in segmental volume of ≥5% indicates a significant difference between the volumes of each segment, while a difference of <5% denotes equal segmental volumes.

*References*

1. Eguchi T, Sato T, Shimizu K. Technical advances in segmentectomy for lung cancer: a minimally invasive strategy for deep, small, and impalpable tumors. Cancers (Basel) 2021; 13.
2. Zhou X, Hayashi T, Hara T et al. Automatic segmentation and recognition of anatomical lung structures from high-resolution chest CT images. Comput Med Imaging Graph 2006; 30: 299-313.

3. Nakao M, Omura K, Hashimoto K et al. Novel three-dimensional image simulation for lung segmentectomy developed with surgeons’ perspective. Gen Thorac Cardiovasc Surg 2021; 69: 1360-1365.

**Supplemental Table S1.** Comparison of the distribution of the right upper lobe bronchial branching patterns between our study and a previous study.

| **Bronchial branching pattern** | **Our study (n=303)** | **Nagashima et al. (n=263)** |
| --- | --- | --- |
|  | **n (%)** | **n (%)** |
| **Typical trifurcated** |  |  |
| B^1^/B^2^/B^3^ | 195 (64) | 116 (44) |
| **Bifurcated non-defective** |  |  |
| B_1_+B_2_/B_3_ | 49 (16) | 38 (14) |
| B_1_+B_3_/B_2_ | 10 (3) | 23 (9) |
| B_1_/B_2_+B_3_ | 8 (3) | 16 (6) |
| **Bifurcated defective** |  |  |
| BX_1_a+B_2_/BX_1_b+B_3_ | 19 (6) | 13 (5) |
| B_1_+BX_2_a/B_3_+BX_2_b | 5 (2) | 6 (2) |
| B_1_ +BX_3_a/B_2_+BX_3_b | 2 (1) | NR |
| **Atypical trifurcated** |  |  |
| BX^1^a/B^2^/BX^1^b+B^3^ | 6 (2) | NR |
| BX^1^a+B^2^/BX^1^b/B^3^ | 1 (0.3) | NR |
| BX^1^a/B^2^/BX^1^b+B^3^ | 3 (1) | NR |
| B^1^/BX^2^a/BX^2^b+B^3^ | 2 (0.7) | NR |
| **Unclassified** | 3 (1) | 49 (19) |


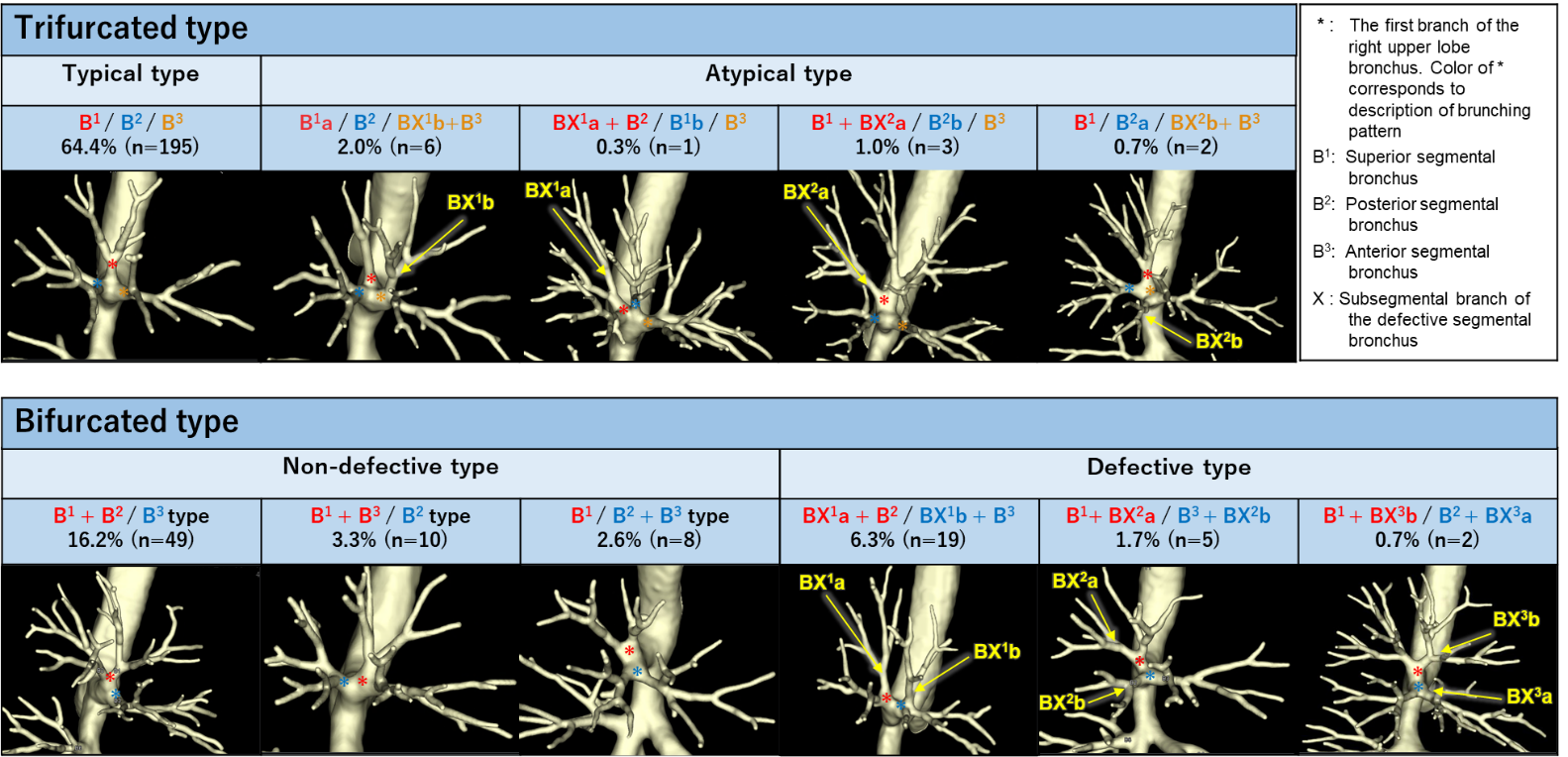
**Supplemental Figure S1.** Detailed classifications of the RUL bronchus.

**Supplemental Figure S2.** Branching pattern of the displaced type (1.0% [n=3]: B^1^ displaced type, 0.3% [n=1]; B^3^ displaced type, 0.7% [n=2]).


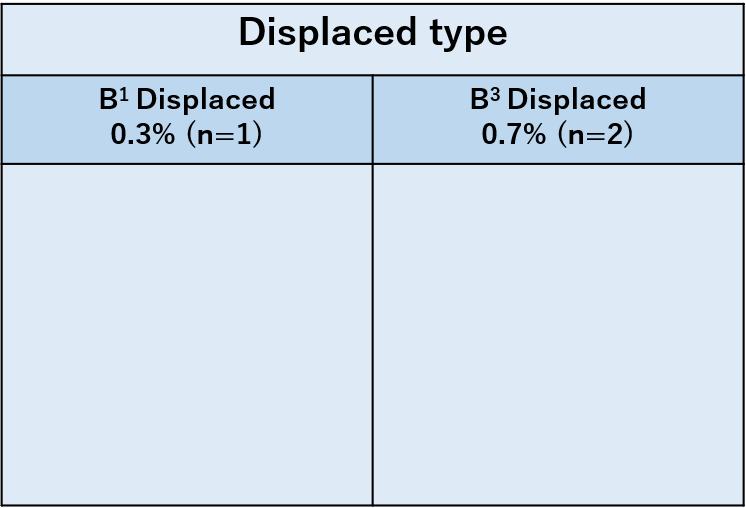

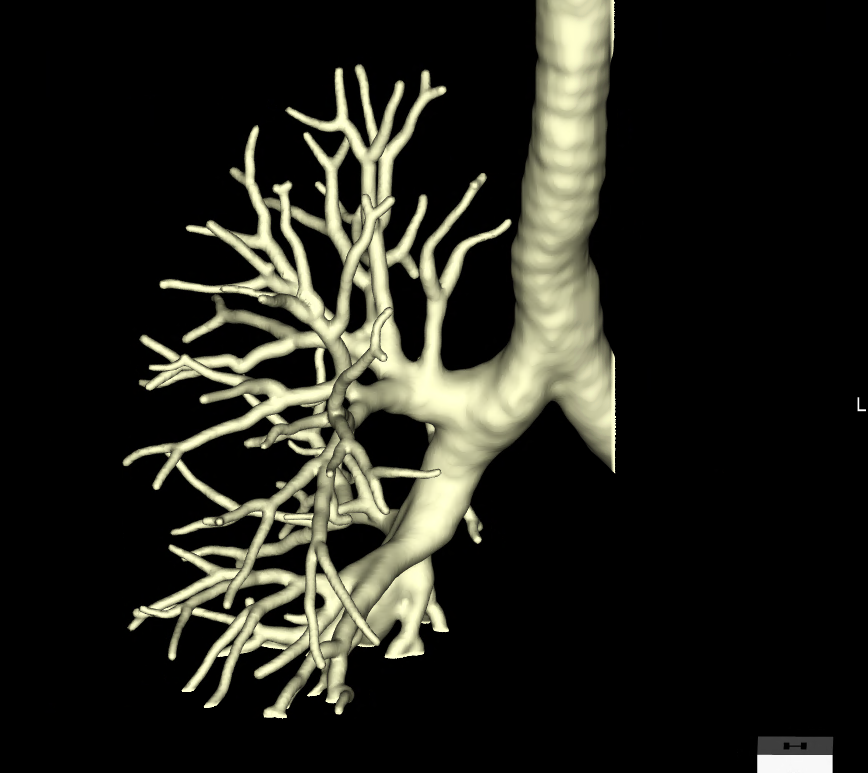


**B^3^**

**B^2^**

**＊**

**＊**

**B^1^**

**＊**

**B^1^b**

**＊**


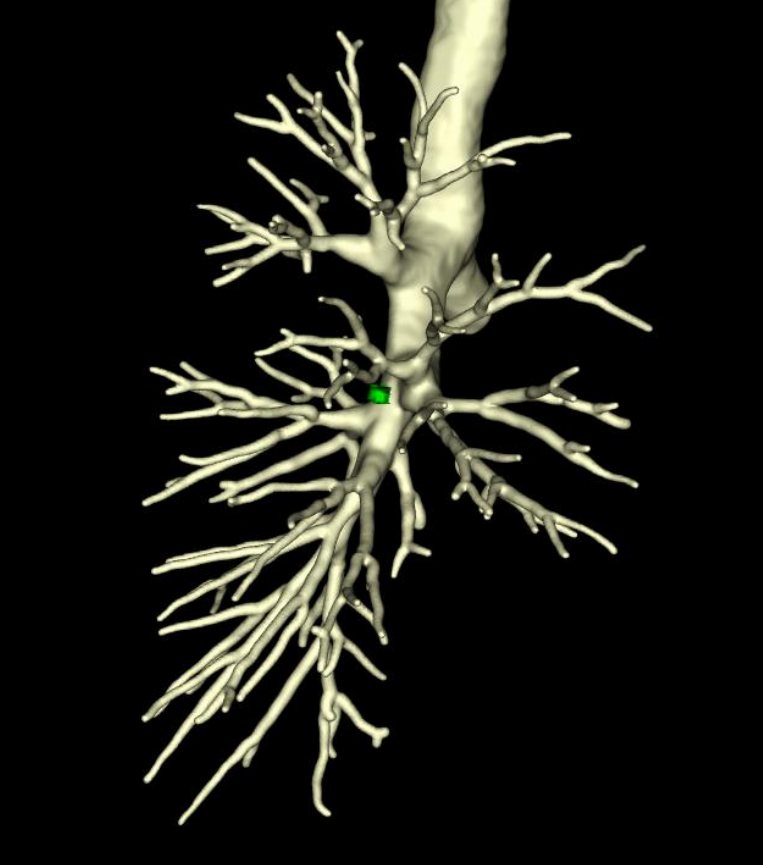


**Supplemental Figure S3.** Illustration of a representative B^1^a/B^2^/BX^1^b+B^3^ bronchial branching pattern.


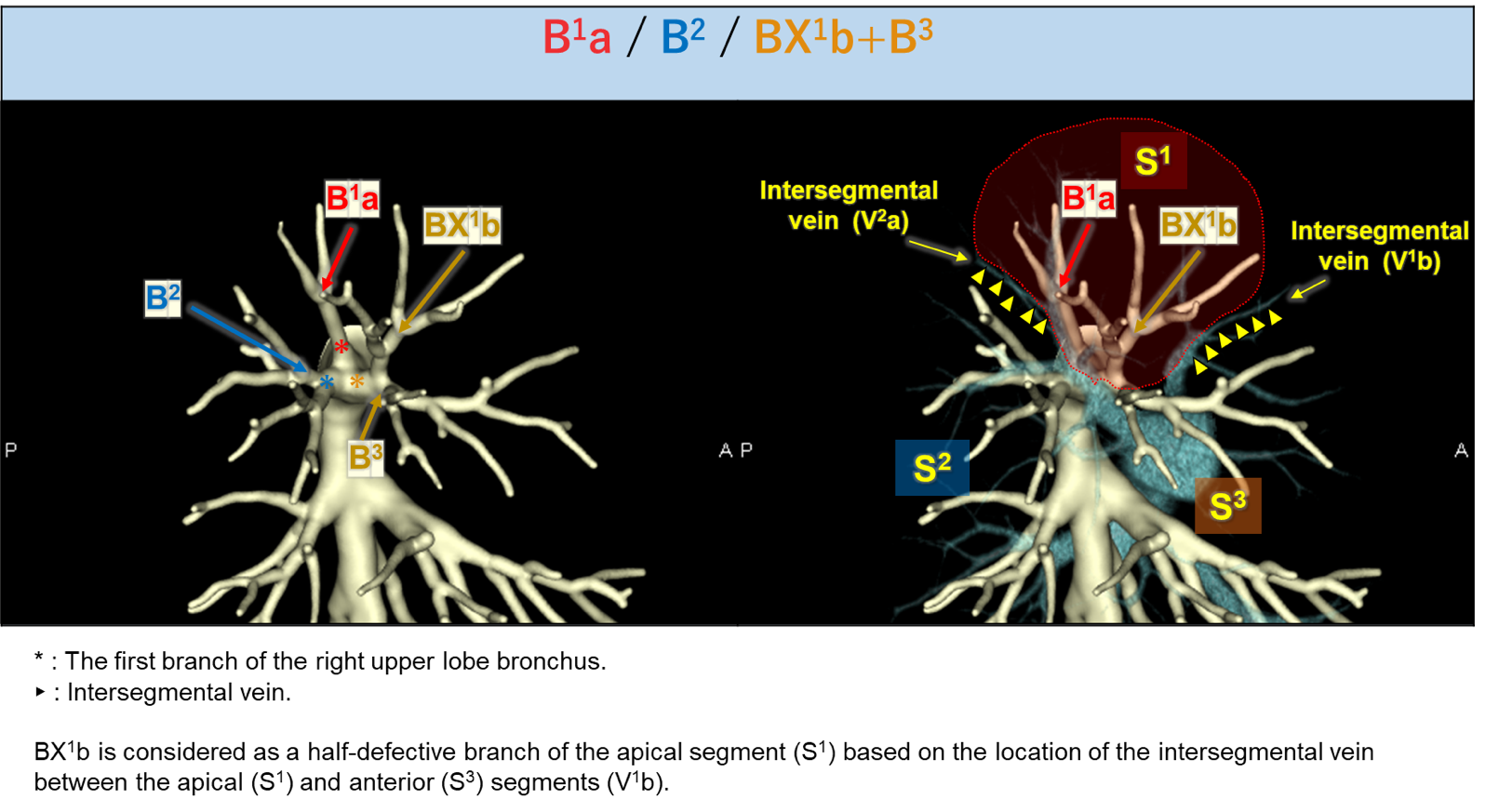


**Supplemental Figure S4.** “Mega S3,” which occupies more than half of the right upper lobe. V1b is the intersegmental vein between S^1^ and S^3^, and V2c is the intersegmental vein between S^2^ and S^3^. The S^3^ volume occupies 61.6% of the right upper lobe in this case.


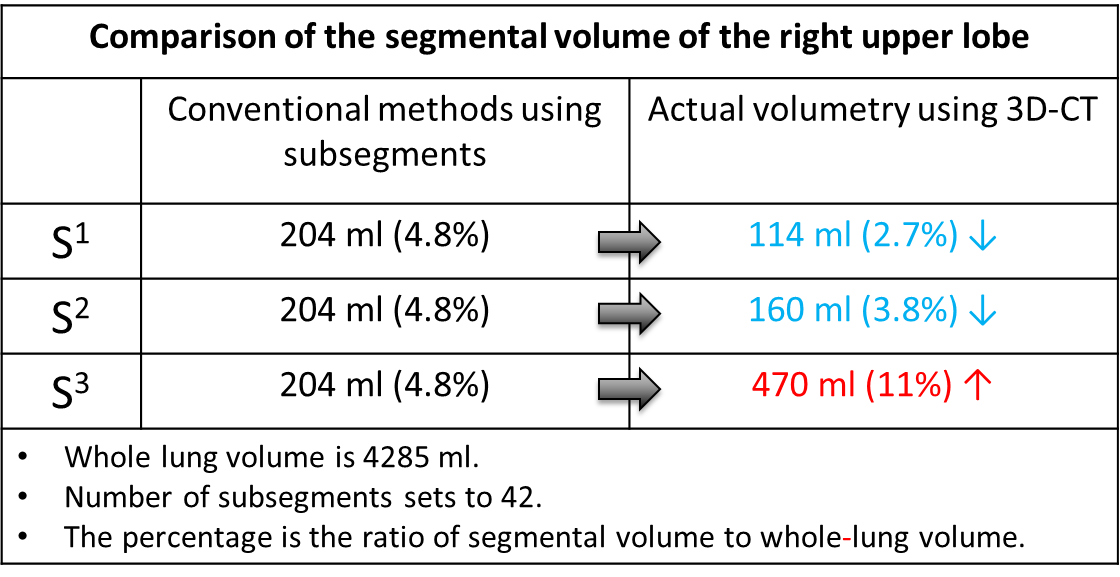


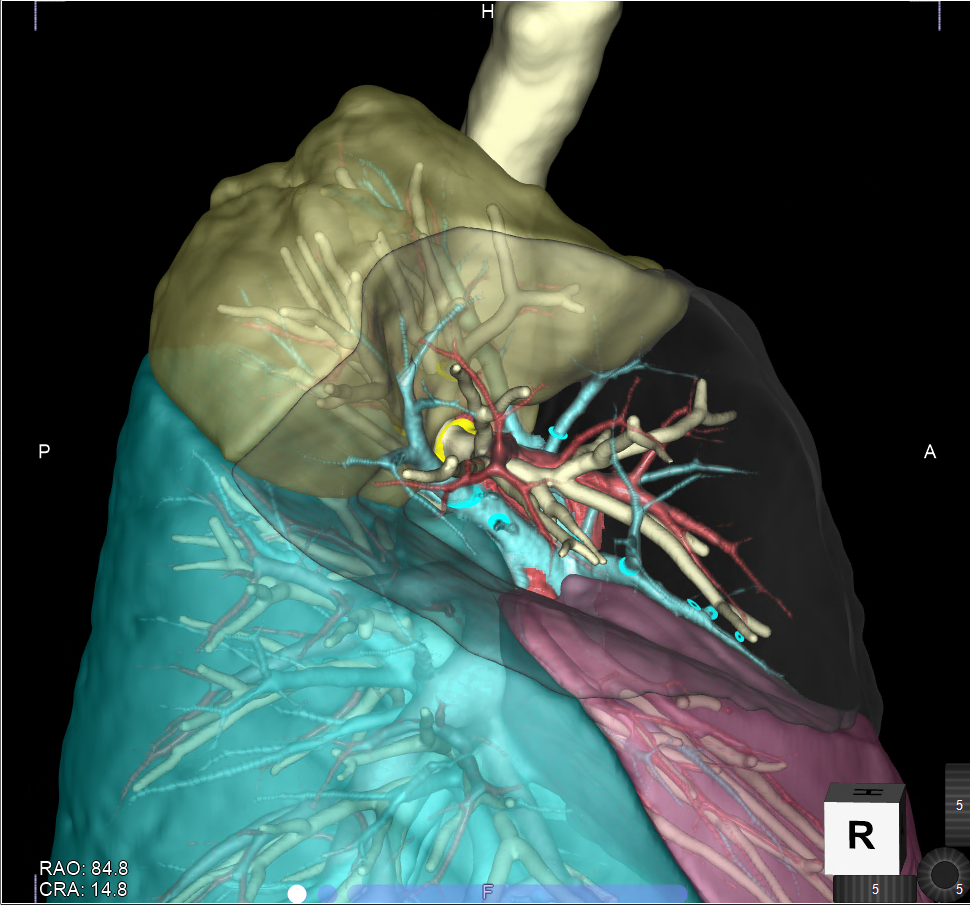


**V^2^c**

**V^1^b**

S^1^: 114ml

S^2^: 160ml

S^3^: 470ml

**Supplemental Figure S5.** Comparison of a 3D image and an image produced by REVORAS.


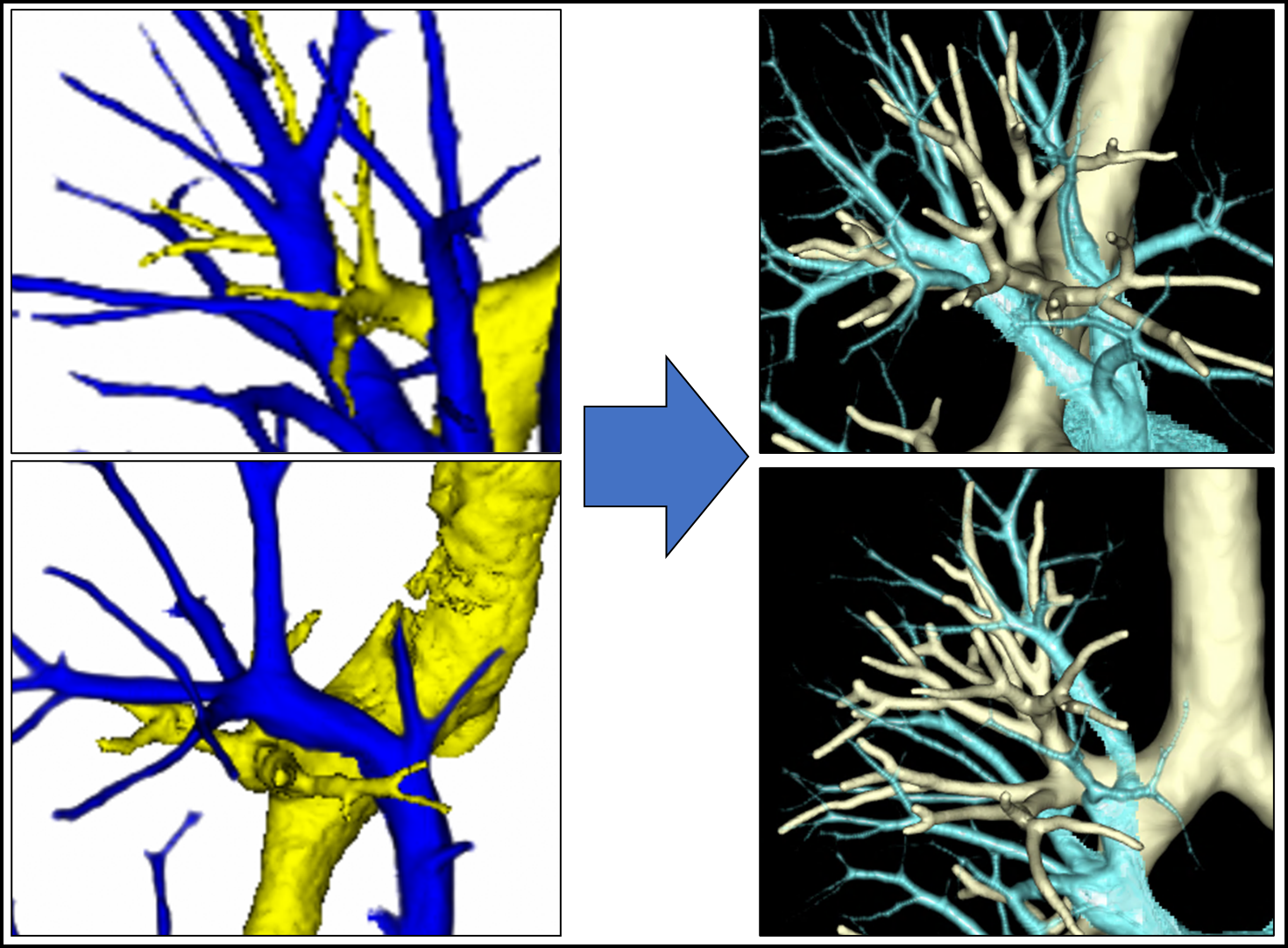

Supplement: ivad136_Supplementary_Data [file ivad136_Supplementary_Data.docx]
